# Ursolic Acid Hydrazide Based Organometallic Complexes: Synthesis, Characterization, Antibacterial, Antioxidant, and Docking Studies

**DOI:** 10.3389/fchem.2018.00055

**Published:** 2018-03-12

**Authors:** Muafia Jabeen, Sajjad Ahmad, Khadija Shahid, Abdul Sadiq, Umer Rashid

**Affiliations:** ^1^Department of Pharmaceutical Chemistry, Riphah Institute of Pharmaceutical Sciences, Riphah International University, Islamabad, Pakistan; ^2^Department of Pharmacy, University of Malakand, Chakdara, Pakistan; ^3^Department of Chemistry, COMSATS Institute of Information Technology, Abbottabad, Pakistan

**Keywords:** ursolic acid, triterpenoid, metal complex, hydrazine, antioxidant, antibacterial, molecular docking

## Abstract

In thecurrent research work,eleven metal complexes were synthesized from the hydrazide derivative of ursolic acid. Metal complexes of tin, antimony and iron were synthesized and characterized by FT-IR and NMR spectroscopy. The antibacterial and antioxidant activities were performed for these complexes, which revealed that the metal complexes synthesized are more potent than their parent compounds. We observed that antioxidant activity showed by triphenyltin complex was significant and least activity have been shown by antimony trichloride complex. The synthesized metal complexes were then evaluated against two Gram-negative and two Gram-positive bacterial strains. Triphenyl tin complex emerged as potent antibacterial agent with MIC value of 8 μg/ml each against Shigellaspp, *Salmonella typhi* and *Staphylococcus aureus*. While, the MIC value against *Streptococcus pneumoniae* is 4 μg/ml. Computational docking studies were carried out on molecular targets to interpret the results of antioxidant and antibacterial activities. Based on the results, it may be inferred that the metal complexes of ursolic acid are more active as compared to the parent drug and may be proved for some other pharmacological potential by further analysis.

## Introduction

Ursolic acid (3β-hydroxy-urs-12-ene-28-oic acid, UA) is a triterpenoid present in many medicinal plants, such as *Eriobotrya japonica, Rosmarinus officinalis, Ocimum sanctum, Melaleuca leucadendron, Piper betle, and Glechomahederaceae* (Liu, 1995). Ursolic acid has been reported with excellent biological potential. It exerts anti-inflammatoryeffects by inhibiting arachidonate metabolism (Baricevic et al., 2001) and also increasesthe nitric oxide synthase activity in endothelial cells (Shin et al., 2004). UA acts as an inhibitor of PTP1b with 10-times greater potency, topromote insulin receptor phosphorylation and glucose uptake in L6 myotubes (Gum et al., 2003). Ithas strong hepatoprotective activity against ethanol. Balanehru et al have shown that UA has strong protective effect against free radical damage in heart than in liver *in*-*vitro* condition (Balanehru and Nagarajan, 1992). It also play a vital role in suppressionofaberrant crypt foci (ACF) formation and have been noted to be protective against colon carcinogenesis (Furtado et al., 2008). Ursolic acid and its derivatives have been reported with antibacterial, antioxidant, anticancer and anti-ulcer potentials by different research groups (Farina et al., 1998; Wolska et al., 2010; do Nascimento et al., 2014; Kalani et al., 2014). Hydrazine, hydrazone and its other analogs are potential group of compounds for various pharmacological potentials. Being a reactive functionality, the acid derivatives of hydrazine are widely synthesized (Audrieth et al., 1954). The most prominent activity reported with hydrazine and its related compounds is the free radicals scavenging effect (Gürkök et al., 2009; Belkheiri et al., 2010; Zhong et al., 2010; Musad et al., 2011; Yilmaz et al., 2012). However, various researchers have also explored the antibacterial and antifungal potentials of such nitrogenous compounds against various microbial strains (Parodi et al., 1981; Akbas and Berber, 2005; Sönmez et al., 2006; Salimon et al., 2010). The hydrazine derivatives have also potential effect as a chemotherapeutic agent for the management of Tuberculosis (Robitzek et al., 1952; Selikoff et al., 1952). Like many other synthetic and natural bioactive compounds (Sadiq et al., 2015; Shah et al., 2015; Zeb et al., 2017), UA has been included in important medicinal compounds and investigational studies are being carried out in various aspects (Ziegler et al., 2004; Liu, 2005).

The metal complexesplay vital roles in catalysis, materials synthesis and photochemistry (Greenwood and Earnshaw, 2012). Tin, a metallic element strongly affects the biochemical property of organo-tin compounds (Jastrzebski and Van Koten, 1993). Therefore,organo-tin (IV) complexes, organo-tin (IV) carboxylates have potent antifungal, antibacterial, and antitumor activities (Davies and Smith, 1980). Commonly, triorgano-tin (IV) compounds are more active than their di and monoorgano-tin (IV) analogs, this activity related to binding of proteins (Blunden and Evans, 1990). Copper (Cu) hasanticancer activity, particularly used against breast cancer cells and proliferative cells (MCF7) (Yousefi et al., 2011). CopperII complexes display cytotoxic activity against human acute lymphoblastic leukemia CCRF-CEM cells and colon adenocarcinoma HT-29 cells an also used as antiseptic (Easmon et al., 2001). Copper is involved in hemoglobin formation, drug/xenobiotic metabolism, carbohydrate metabolism, catecholamine biosynthesis and in cross-linking of collagen, elastin and hair keratin as well as in the antioxidant defense characteristics because it is present in many metallo-enzyme (Ergene et al., 2010). Similarly, Hydrazine (diazane) is an inorganic compound with the formula N_2_H_4_, possessing H_2_N-NH- subunit and constitute an important class of compounds for new drug development. Hydrazides are the derivatives of hydrazine with at least one acyl group [R-C(=O)-NH-NH_2_]. Medicinal and drug discovery chemists are in constant research to synthesize these compounds as target structures and to embark their various biological potentials (Kim et al., 1999).

In this research work, hydrazine derivative of ursolic acid was synthesized as a ligand and then different metal complexes of the ligand were synthesized. Furthermore, the antibacterial and antioxidant activities of the complexes were carried out. Additionally, we have supported our *in-vitro* results with *in-silico* studies.

## Materials and methods

### Synthesis of ligand

Direct etherification method was used for the synthesis of esters. In a flask, mixture containing 0.2 moles of UA, conc. sulphuric acid (0.4 mol) and 0.2 ml of ethanol were taken and the mixture was allowed to reflux for 3 h. Surplus ethanol was distilled off under low pressure employing rotary apparatus and allowed the mixture to cool at room temperature. It was then filtered and the residue obtained was dried over silica gel in a desiccator.

Hydrazine hydrate (0.1 g) and ethyl ester of UA (0.002 mol) were taken in a flask. A small volume of ethanol was mixed for the formation of clear reaction mixture. For 3–5 h the contentwas allowed to reflux. The evaporation of excess ethanol was carried out via rotary evaporator to get the productin 85% yield.

### Synthesis of metal complexes

#### Procedure for the synthesis of copper complex (LCu)

Copper complex was synthesized by using 2.0 mmol of hydrazide of UA (ligand, UL) in 30 mL of (**solution 1**). In another flask, copper acetate monohydrate [Cu (CH_3_COO)_2_.H_2_O, 2.0 mmol] was mixed in 20 mL of methanol (**solution 2**). Solution 1 was added with solution 2. The reaction mixture was maintained at pH of 7.0 ± 0.05 with the help of triethylamine. The mixture was stirred for 4 h at room temperature and resultant mixture was allowed to stay overnight in refrigerator. A green colored precipitates was filtered by filtration, and dried over silica gel in a desiccator. The pure product was obtained in 60% yield.

#### Procedure for the synthesis of copper complex (LZn)

Ligand (UL, 1.0 mmol) and zinc acetate dihydrate [Zn(CH3COOH)_2_.2H_2_O, 1.0 mmol] were mixed in equal volume of methanol (25 ml) separately, after mixing pH of solution was maintained at 7.5 with the addition of 0.1% potassium hydroxide in methanol. The mixture was then refluxed for 3 h. Finally, the mixture was filtered and dried over silica gel to obtain a light yellow product in 65% yield.

#### Procedure for the synthesis of iron complex (LFe)

A solution of Ligand (1.0 mmol) in ethanol (5.0 ml) was mixed and then solution of FeCl_3_ (1.0 mmol) was added to it. The mixture was refluxedfor 50 min with continuous stirring. The obtainedcolored solution was reserved at room temperature. In the filtrate, the obtained product was dried with silica gel in a desiccator. The pure product was obtained in 65% yield.

#### Procedure for the synthesis of antimony complex (LSC and LSB) by using antimony trichloride/bromide

Ligand (1.0 mmol) in 15 ml of acetonitrile and solution of SbCl_3_/ SbBr_3_(1.0 mmol) in 15 ml of acetone was added according to molar ratio of 1:1. The mixture was stirred for 30 min. The mixture was filtered and allowed to set for crystallization at room temperature. The desire product was dried in silica gel desiccator. The pure product was obtained in 73% yield (from SbCl_3_) and 72% (from SbBr_3_).

#### Procedure for the synthesis of tin complexes (LDM, LTM, LDB, LTB, LDP, LTP)

A ligand's solution (1.0 mmol) was prepared in 50 ml of toluene (dry). Then triethylamine (1.0 mmol) was added to the ligand solution and refluxed for 3 h. After-wards, the reaction mixture was cooled down to room temperature. Organotin compounds (1.0 mmol) was added to a flask with continuous stirring and refluxed again for 8–10 h. After reflux completion, the mixture was filtered. The solvent was evaporated through rotary evaporator. The residue was obtained and re-crystallized with the help of chloroform (Atta-ur-Rahman et al., 2001). Dimethyltin complex (LDM) was obtained in 65% yield. While, trimethyl complex (LTM), dibutyl (LDB), tributyl (LTB), diphenyl (LDP) and triphenyltin complex (LTP) were obtained in 67, 70, 72, 60, and 65% yield respectively.

### Solubility method

First of all apparent solubility of all the synthesized metals were determined. A weighed amount of the metal complexes were dissolved in measured amount of solvent and clarity/turbidity was observed. Another experimentally simple method for the solubility determination was carried out as described by Harle et al. (2003) using Buchner funnel. Methanol, ethanol, DMSO, chloroform and toluene were used for this study. For good solubility, the threshold value of <0.1/100 mL with a clear colored solution in the given solvent was considered.

### Antibacterial assay

The antibacterial activity of ursolic acid, the ligand and their metal complexes were determined against various bacterial strains using well diffusion assay. The tested strains include Gram negative like, *Escherichia coli, Salmonella typhi*, and Shigella species, and Gram positive strains include *Staphylococcus aureus*and *Bacillus subtilus*.

Aliquot of nutrient broth (10 ml)was made in this method which was inoculated with bacteria at 37 (±1)°C for 24 h. Broth culture (0.6 ml) of the test organism was added in a Petri dish via a sterile pipette. This was also included with 20 ml of agar having 0.2 ml culture and was heated at 45°C. This was suspended properly and was added into a sterile Petri dish. Plate of each organism was obtained in a similar way. The agar was allowed to dry/solidify and with the help of a sterile cork borer, the required numbers of holes (10 mm) were cut. The holes were designed and distributed properly, one in the center and others in the periphery. The agar plugs were removed.

Solution of the ligand and metal complexes were made individually having concentration of 1.0 mg/ml. With the help of micropipette, test sample of 100 μl was added in a solvent. The holes were labeled and the test samples were added properly. A solution of tetracycline (1.0 mg/ml) was used as positive control. The plates are allowed to stay at room temperature for 2 h for proper diffusion of the sample and incubated at 37 ± 1°C for 24 h. Hence antibacterial activity was determined by calculations of the zone of inhibition in millimeters (Sadiq et al., 2016).

### Determination of antifungal activity by agar tube dilution assay

The antifungal activity of UA, ligand and their metal complexes were evaluated against six (6) fungal strains. The fungal strains used were*Candida alibican, Tricophytonlongifusus, Microsporumcanis, Asperigillusflavus, Fusarium solani*, and Bipol using agar tube diffusion method. Sabouraud 4% Glucose Agar was mixed in distilled water with stirring to make sabouraud dextrose agar and heated. Prepared media was added into test tubes and autoclaved at for 15 min at 121°C. Autoclaved tubes wereheated upto 50°C. Solution of each test sample (20 μg/ml) was made in sterile DMSO. Ketoconazole in the same concentration, i.e., 20 μg/ml served as control drug. With the help of a micropipette, 100 μl solutions of test sample and control sample was added into different tubes of non-solidified agar media. The tubes were allowed to solidify at room temperature. Each of the tube was inoculated with inoculums of 4 mm diameter piece which was obtained from 7 days old fungal strains' culture. Then all tubes were incubated at optimum temperature, i.e., 28–30°C for 7–10 days. The controlled humidity (40–50%) was achieved with the help of an open pan of water in the incubator. During incubation, the cultures were observed at least twice a week. After 7–10 days of incubation,minimum inhibitory concentration (MIC in μg/mL)MIC was calculated as the lowest concentration of the extract inhibiting the growth of bacterial strainto obtain a final concentration ranging from 256 to 0.16 μg/ml. The assay was performed as triplicate analysis (Irobi et al., 1996).

### Determination of antioxidant activity

The DPPH assay (DPPH free radicals) was used to find out the anti-radical properties of the samples (Ahmad et al., 2015). DPPH solution was prepared by adding 3.2 mg of it into 100 ml methanol (82%). Into a glass vial, 2,800 μl of DPPH solution was added followed by 200 μl of test sample. The serial dilutions were prepared in concentration of 1,000, 500, 250, 125, and 62.5 μg/ml.

The mixture was stirred and allowed to stay at room temperature in dark for 1 h. UV-Visible Spectrophotometer (Shimadzu 1,800) was used for recording of absorbance. A mixture of 82% methanol (2,800 μl) and 200 μl methanol was used as blank. Whilemethanol 200 and 2,800 μl of DPPH solution was used as a control. The test was repeated three (3) times. The % inhibition was find out by using the given formula.

$$\begin{array}{l} \text{Scavenging effect }\left(\%\right)\;=\;\frac{{\text{A}}_{\text{c}}-{\text{A}}_{\text{s}}}{{\text{A}}_{\text{c}}}\times \;100 \end{array}$$

Where A_c_ is the control absorbance and A_s_ is the test sample absorbance.

### Docking studies

Docking studies were carried out using Autodock 4.2. Three dimensional (3D) crystal structures of the enzymes retrieved from Protein Data Bank (PDB). For the study of antioxidant activity, molecular docking studies were carried out human peroxiredoxins (PrxV, PDB code 3MNG) with a competitive antioxidant inhibitor 1,2-dithiane-4,5-diol (DTT) was retrieved from Protein Data Bank (PDB). While, for study of the binding orientation for the antibacterial study, penicillin binding protein (PBP) with PDB code 2EX9 with penicillin V as co-crystallized ligand was retrieved.

The structures of the metal complexes were drawn using Marvin sketch 16.5.2. The 3D structures of these complexes were optimized using prepare ligand module in Autodock 4.2. The parameter file of AutoDock was modified to incorporate metalVan der Waals and other needed parameters which were obtained from the AutoDock website. For enzyme downloaded from PDB, solvation parameters and Kollman charges for all the atoms were assigned. AutoDock Tools (ADT) were used to create PDBQT file for both ligand and enzyme. A grid parameter file was generated using ADT. A cubic grid box of 60 Å (x, y, z) (for 3MNG) and a cubic grid box of 45 Å (x, y, z) (for 2EX9) with a spacing of 0.375 Å was created.

The reliability of docking program was validated by using re-docking method. In both cases the co-crystallized ligands were re-docked into the active site of the downloaded enzymes. Root Mean Square (RMSD) was then calculated between the co-crystallized and re-docked poses. In both case, the RMSD value are within the threshold limit. In all cases, RMSD value of <2.0 Å is considered as accurate in predicting binding orientation of ligand. The binding poses were studied using discovery studio visualizer.

## Results and discussions

### Physical data and solubility

**Figure 1:**
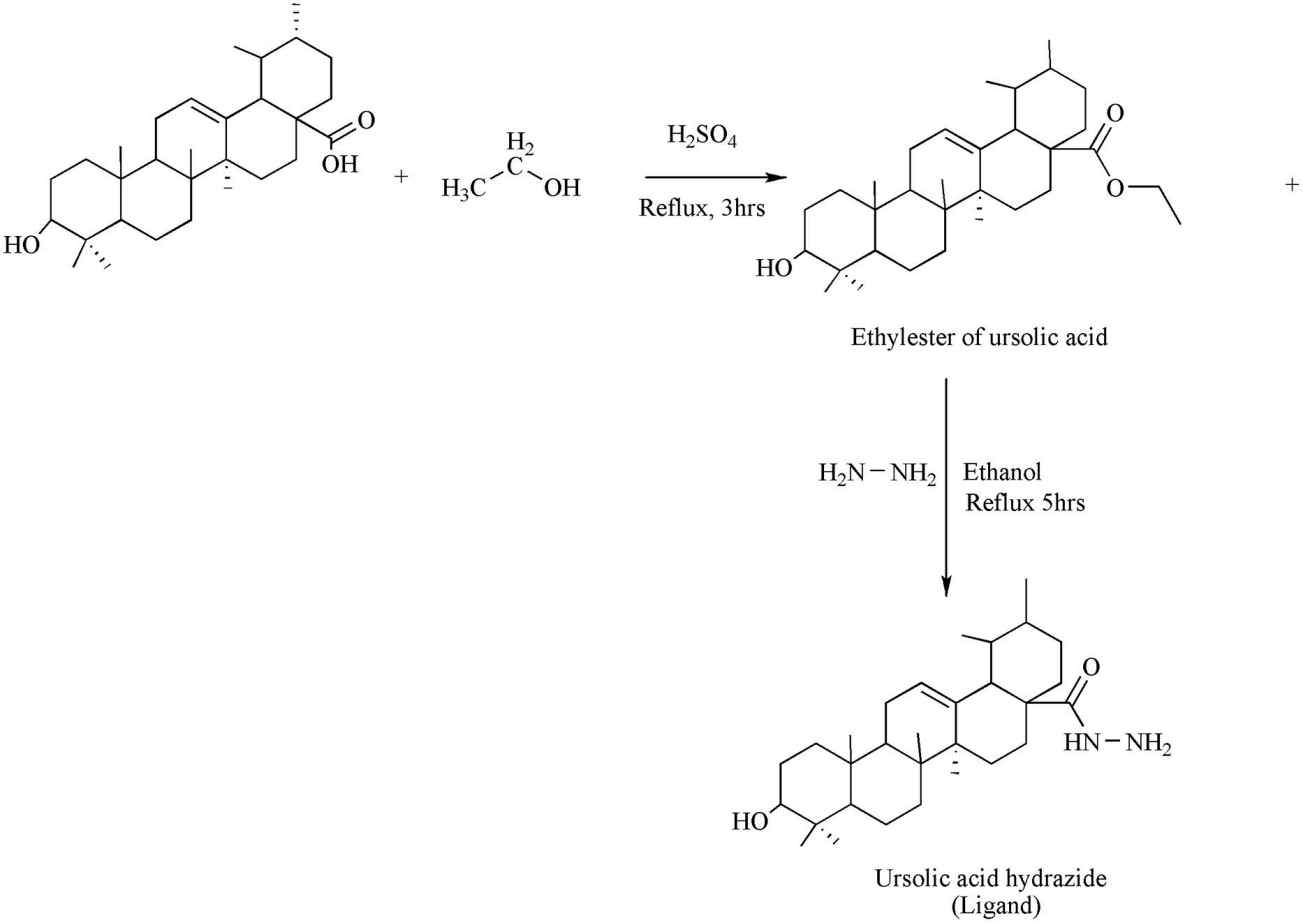
Synthesis of Ligand.

**Figure 2:**
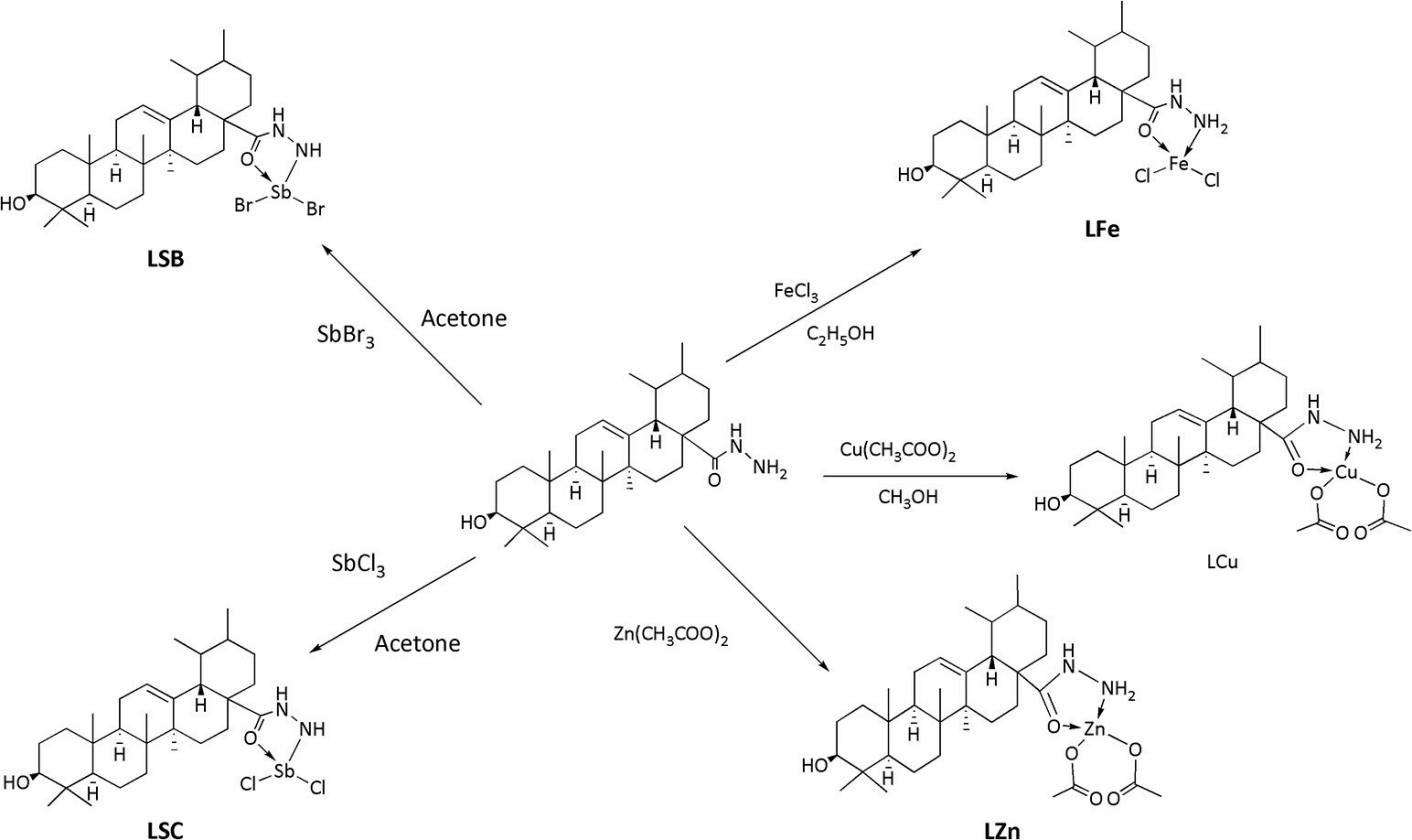
Synthesis of Cu, Fe, Zn and Sb complexes.

**Figure 3:**
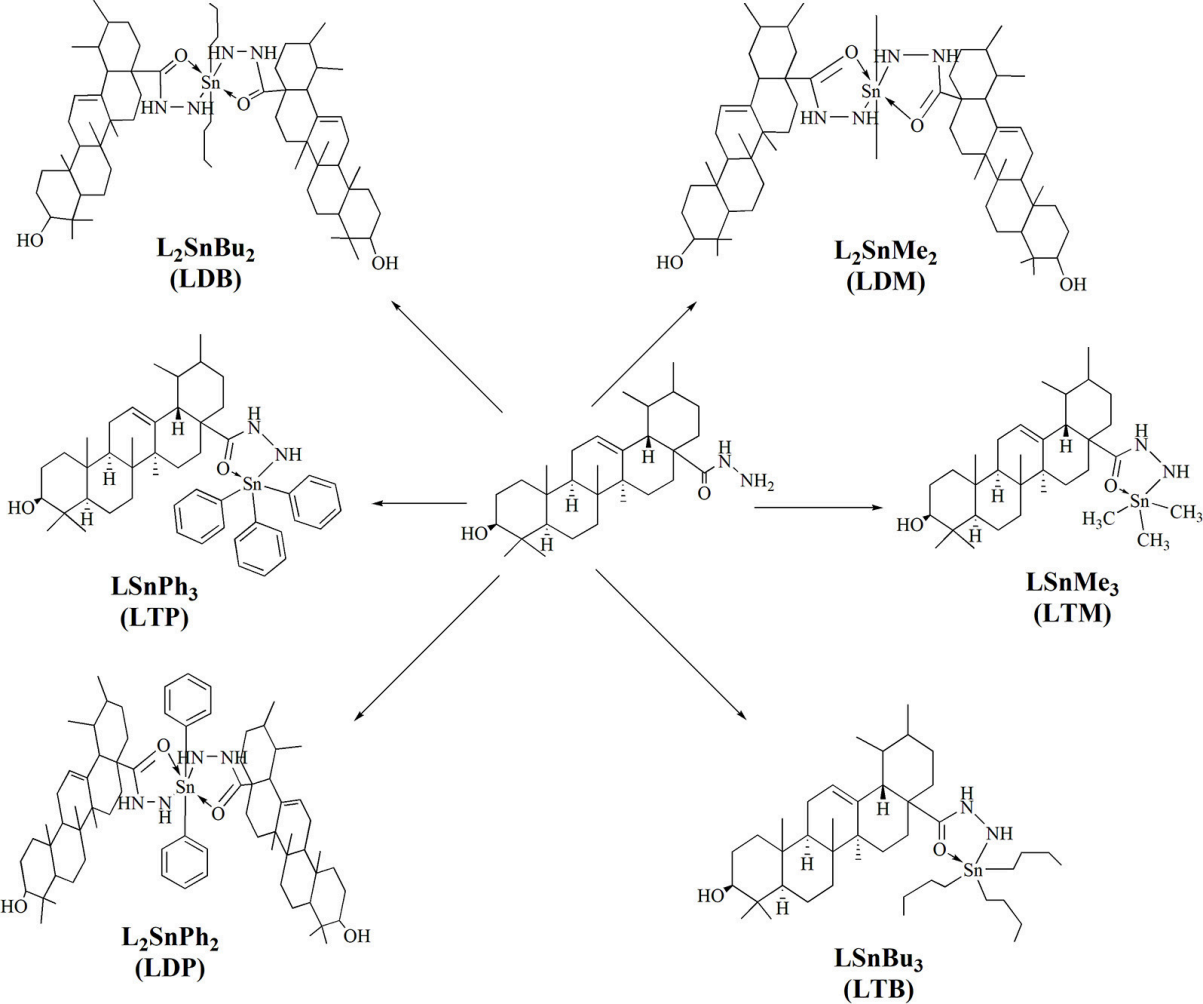
Synthesis of tin complexes.

The synthesis of the ligand is shown in Figure 1 while its metal complexation is provided in Figures 2, 3. The physical data and solubility of ligand and metals complexes have been presented in the Tables 1, 2 respectively. The abbreviations of various complexes have also been mentioned in these tables.

**Table 1:** Physical data of ligand and their metal complexes.

**S. N**	**Identity codes**	**Mol. Formula**	**Formula weight (g/mol)**	**Isolated yield (%)**	**Melting point (°C) & color**
1	UL	Ligand	471	85	115, White
2	LCu	LCu(CH_3_COO)_2_	652	60	80, light Gray
3	LZn	LZn(CH_3_COO)_2_	690	65	155, White
4	LFe	LFeCl_2_	597	65	160, off White
5	LSC	LSbCl_2_	663	73	180, off White
6	LSB	LSbBr2	752	72	170, off White
7	LDM	LMe_2_SnCl_2_	690	65	100, off White
8	LTM	LMe_3_SnCl	670	67	80, Yellowish
9	LDB	LBu_2_SnCl_2_	774	70	95, White
10	LTB	LBu_3_SnCL	796	72	130, Yellowish
11	LDP	LPhe_2_SnCL_2_	815	60	140, Yellowish
12	LTP	LPhe_3_SnCl	856	65	180, Grayish

**Table 2:** Solubility of the ligand and its metal complexes.

**S. N**	**Compound**	**Methanol**	**Ethanol**	DMSO^*^	**Chloroform**	**Toluene**
1	UL	Yes^**^	No	Yes	Yes	Yes
2	LZn	No	No	Yes	Yes	Yes
3	LCu	No	Yes	Yes	No	Yes
4	LSB	Yes	Yes	Yes	Yes	Yes
5	LSC	Yes	Yes	No	No	No
6	LFe	Yes	Yes	Yes	Yes	Yes
7	LTP	No	No	Yes	Yes	Yes
8	LDP	Yes	Yes	No	No	No
9	LTB	Yes	Yes	No	No	No
10	LDB	Yes	Yes	No	No	No
11	LTM	Yes	Yes	No	No	No
12	LDM	Yes	Yes	Yes	Yes	Yes

* *Dimethylsulfoxide*.

** *Compounds with solubility < 0.1 mg/100 mL*.

### IR spectral analysis and IR spectrum of dipheyltin complex

**Figure 4:**
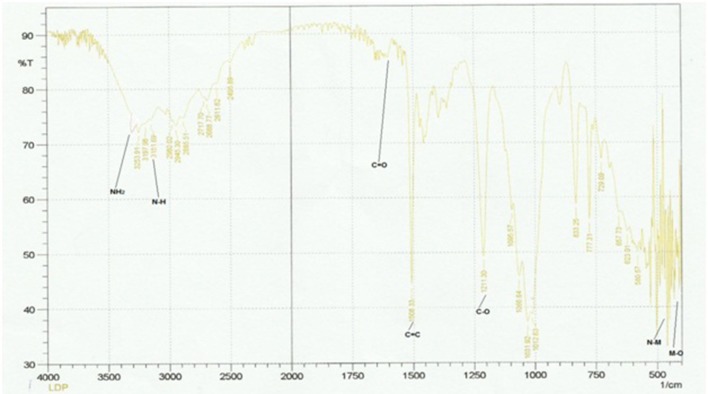
IR spectrum of diphenyltin complex.

The Figure 4 and Table 3 shows the most important bands of FT-IR spectra recorded for all the ligands and complexes. The attachment of metals can be found out by the absence of primary stretch in the metal complexes from the ligand. The metal complex can be confirmed from the appearance of new bands in FT-IR, which are not present in the ligand's spectrum. The band of NH has been shifted to the lower band area i.e., about 30 cm^−1^ due to coordination. The NH groups showed no significant shift in its fundamental shift. This confirms that negligible interaction is there between metal complexes and N=O group. The IR investigation reveals that designed molecular structures of metal complexes go parallel with the observations from the experiments. The NH_2_, C=O, C-O, NH and C=C peaks can be seen in the ligand's spectrum. The NH_2_, NH, C-O and C=C stretches have been recorded in 3,360, 3,200, 1,215, and 1,650 cm^−1^ respectively. The new complex formation can also be confirmed from the new peaks in zinc complex spectrum. The M-O and M-N can be observed in 400 and 594 cm^−1^ respectively. The NH2, C=C aromatic, C-C, C=O and NH peaks have also been shown in the spectrum. The peaks of M-O and M-N in the diphenyl tin complex spectrum can be seen at 400 and above 450 cm^−1^ respectively. The C-O, C=C, C=O, NH2 and NH peaks appear at 1,211, 1,650 to 1,700, 1,508, 3,300, and 3,151 cm^−1^ respectively. So the molecular structure of diphenyl tin complex was confirmed from the data obtained.

**Table 3:** IR spectral analysis of ligand and its metal complexes.

**Complex**	**Frequency(cm**^**−1**^**)**						
	**C=C**	**C=O**	**NH_2_**	**ÑH**	**ÑM**	**$\tilde{\text{M}}$O**	**$\tilde{\text{C}}$O^s^**
Ligand(UL)	1,650	1,508	3,358^s^, 1,637^b^	3,209	–	–	1,215
LTP	1,680	1,508	3,360	3,197	570	435	1,047
LDP	1,695	1,508	3,253^s^, 1,635^b^	3,151	580	483	1,031
LTB	1,680	1,508	3,365^s^	3209	501	401	1,211
LDB	1,650	1,550	1,597^b^	2,951	513	410	1,031
LTM	1658	1561	1608^b^	3,034	545	651	1029
LDM	1,658	1,591	1,614^b^	3,240^s^	540	–	1033
LFe	1,633	1,506	1,633^b^	3,209^1^	511	401	1,109
LCuSO_4_	1,650	1,508	3,354^s^, 1,602^b^	–	495	414	1,012
LZn(Ac)_2_	1625	1539	3348	3,257	491	400	1,012
LSbCl_2_	1,650	1,510	3,360^s^, 1,633^b^	3,273	567	416	1,049
LSbBr_2_	1,680	1,513	3,367^s^	–	578	433	1,064

s *Stretching frequency*.

b *Bending frequency*.

### ^1^H NMR spectroscopy

**Figure 5:**
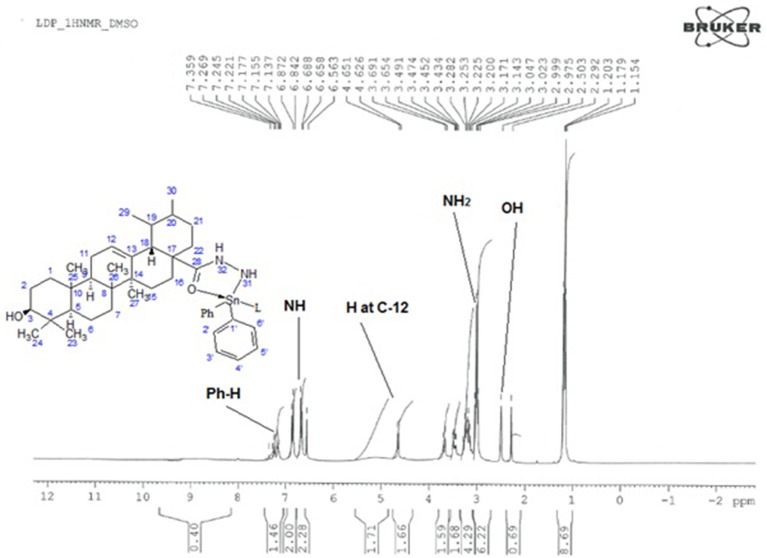
^1^H NMR Spectrum of Diphenyltin complex.

The proposed molecular structure of compounds was confirmed from the peaks in ^1^HNMR spectrum of metal complexes and ligand. The ^1^H NMR data of compounds with various complexes have been presented in Tables 4A,B. The hydrogen of hydroxyl group, NH2 group and phenyl group have been shown at 2.4, 3.4, and 7.2 chemical shifts respectively as shown in Figure 5. So this analysis confirms the structure.

**Table 4A:** ^1^HNMR spectral data of ligand and their metal complexes.

**H No**	**Compound assignment**	**L δ(ppm)**	**LFeCl_2_ δ(ppm)**	**LCu(Ac)_2_ δ(ppm)**	**LZn(Ac)_2_ δ(ppm)**	**LSbCl_3_ δ(ppm)**	**LSbBr_3_ δ(ppm)**
1	a.CH b.CH	1.70 1.28	1.25 1.47	1.70 1.25	1.45 1.25	1.20 1.30	1.23 1.35
2	a.CH b.CH	1.71 1.25	1.71 1.47	1.71 1.38	1.72 1.45	1.40 1.70	1.43 1.71
3	CH	3.11	3.15	3.30	3.16	3.15	3.17
3′	OH	2.40	2.25	2.50	2.50	2.10	2.20
4	–	–	–	–	–	–	–
5	CH	1.29	1.35	1.37	1.38	1.31	1.32
6	a.CH b.CH	1.27 1.73	1.52 1.27	1.23 1.50	1.50 1.25	1.42 1.27	1.43 1.27
7	a.CH b.CH	1.63 1.16	1.47 1.24	1.48 1.25	1.50 1.24	1.28 1.47	1.27 1.46
8	–	–	–	–	–	–	–
9	CH	1.57	1.43	1.43	1.50	1.43	1.41
10	–	–	–	–	–	–	–
11	a.CH b.CH	2.20 1.27	2.01 1.16	2.10 1.75	1.88 1.76	1.70 2.04	1.80 2.13
12	CH	4.70	5.28	5.21	5.11	4.60	4.70
13	–	–	–	–	–	–	–
14	–	–	–	–	–	–	–
15	a.CH b.CH	1.27 1.11	1.31 1.16	1.29 1.13	1.30 1.16	1.10 1.14	1.12 1.13
16	a.CH b.CH	1.25 1.11	1.62 1.36	1.61 1.35	1.50 1.26	1.70 1.31	1.71 1.31
17	–	–	–	–	–	–	–
18	CH	2.49	2.30	2.30	2.50	2.50	2.76
19	a.CH b.CH	1.71	1.63	1.53	1.76	1.70	1.65
20	–	–	1.61	1.45	1.56	1.60	1.65
21	a.CH b.CH	1.39 1.16	1.52 1.26	1.50 1.27	1.51 1.25	1.20 1.24	1.27 1.23
22	a.CH b.CH	1.71 1.41	1.75 1.52	1.75 1.50	1.76 1.25	1.70 1.53	1.71 1.53
23	CH_3_	1.11	1.11	1.11	1.11	1.11	1.11
24	CH_3_	1.10	1.10	1.10	1.10	1.10	1.10
25	CH_3_	1.60	1.15	1.15	1.14	1.16	1.15
26	CH_3_	1.16	1.16	1.16	1.17	1.16	1.18
27	CH_3_	1.28	1.27	1.26	1.25	1.28	1.30
28	CO	1.06	1.05	1.04	1.05	1.05	1.04
29	CH_3_	1.10	1.05	1.05	1.06	1.05	1.06
30	CH_3_	7.90	8.70	7.66	8.80	8.05	8.08
31	CONH	2.31	2.40	2.42	2.50	2.11	2.41
32	NH_2_	4.31	3.40	3.42	3.40	3.31	3.41
1′	OCH_3_	–	–	2.21	2.13	–	–

**Table 4B:** ^1^HNMR spectral data of ligand and their metal (Tin) complexes.

**Compound**		**L δ(ppm)**	**LSnPh_3_**	**L_2_SnPh_2_**	**LSnBu_3_**	**L_2_SnBu_2_**	**LSnMe_3_**	**L_2_SnMe_2_**
**H #**.	**Atom**	**δ(ppm)**	**δ(ppm)**	**δ(ppm)**	**δ(ppm)**	**δ(ppm)**	**δ(ppm)**	**δ(ppm)**
1	a.CH b. CH	1.22 1.18	1.41 1.20	1.43 1.20	1.41 1.20	1.43 1.28	1.46 1.23	1.43 1.20
2	a.CH b.CH	1.21 1.18	1.71 1.47	1.71 1.48	1.70 1.47	1.72 1.47	1.78 1.45	1.72 1.38
3	CH	2.99	3.00	3.17	3.15	3.09	3.30	3.10
3′	OH	3.15	2.50	2.29	2.50	2.51	2.30	2.98
4	–	–	–	–	–	–	–	–
5	CH	1.45	1.38	1.30	1.31	1.33	1.36	1.35
6	a.CH b.CH	1.09 1.01	1.57 1.20	1.06 1.20	1.50 1.20	1.51 1.21	1.57 1.21	1.57 1.21
7	a.CH b.CH	1.22 1.18	1.47 1.20	1.48 1.20	1.40 1.24	1.41 1.25	1.40 1.23	1.41 1.27
8	–	–	–	–	–	–	–	–
9	CH	1.43	1.45	1.43	1.42	1.43	1.46	1.43
10	–	–	–	–	–	–	–	–
11	a.CH b.CH	1.24 1.22	1.26 1.21	2.05 1.70	2.04 1.71	2.05 1.70	2.06 1.78	2.20 1.70
12	CH	5.01	5.21	4.66	4.67	5.13	5.13	4.64
13	C	–	–	–	–	–	–	–
14	C	–	–	–	–	–	–	–
15	a.CH b. CH	1.22 1.19	1.21 1.18	1.43 1.17	1.37 1.12	1.31 1.10	1.31 1.09	1.32 1.08
16	a.CH b. CH	1.22 1.18	1.22 1.18	1.60 1.32	1.62 1.31	1.63 1.30	1.55 1.31	1.54 1.13
17	–	–	–	–	–	–	–	–
18	CH	2.65	2.76	2.31	2.50	2.51	2.76	2.77
19	a.CH b.CH	1.52 1.35	1.62 1.32	1.62 1.61	1.60 1.62	1.61 1.61	1.55 1.60	1.54 1.67
20	–	–	–	1.50	1.51	1.52	1.50	1.51
21	a.CH b.CH	1.35 1.22	1.29 1.20	1.21 1.70	1.20 1.71	1.21 1.71	1.21 1.71	1.21 1.72
22	a.CH a.CH	1.35 1.22	1.29 1.21	1.51 1.11	1.50 1.11	1.52 1.11	1.51 1.11	1.52 1.11
23	CH_3_	0.72	0.70	1.10	1.10	1.10	1.10	1.10
24	CH_3_	0.71	0.73	1.16	1.15	1.14	1.15	1.12
25	CH_3_	0.65	0.65	1.17	1.16	1.17	1.16	1.17
26	CH_3_	0.85	0.86	1.27	1.28	1.29	1.27	1.28
27	CH_3_	1.07	1.06	–	–	–	–	–
28	CO	–	–	1.05	1.06	1.05	1.07	1.08
29	CH_3_	0.65	0.65	0.73	0.73	0.66	0.77	0.74
30	CH_3_	0.71	0.72	7.40	7.25	7.42	7.26	7.27
31	CONH	8.01	7.38	3.7	2.88	2.80	2.31	2.53
32	NH_2_	4.21	3.6	6.88	1.3,1.32, 1.3, 0.93	1.3,1.32, 1.3, 0.93	0.89	0.87
1′	Ph. H	–	7.7	–	–	–	–	–
2′	Butyl and Methyl Hydrogen	–	–	–	1.2,1.22.1.23, 0.83	1.2,1.22.1.23, 0.83	0.67	0.6

### ^13^C NMR spectral analysis

**Figure 6:**
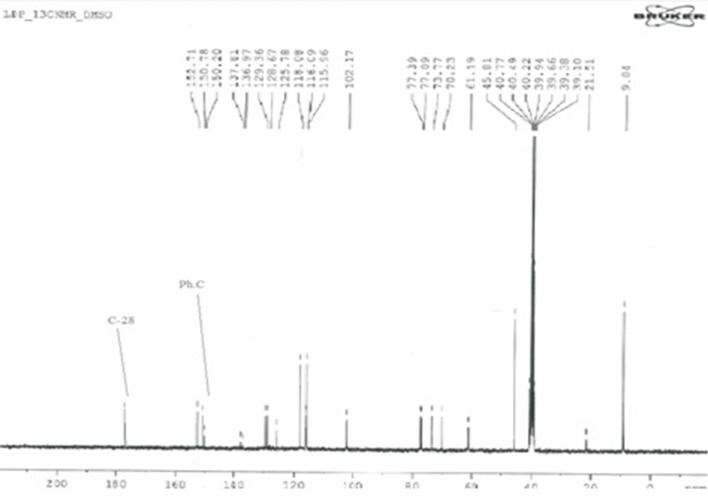
^13^C-NMR Spectrum of diphenyl tin complex.

The ^13^C-NMR spectroscopy of metal complexes and ligand were carried out at 300 MHz on Bruker NMR spectrometer. The solvent used for dissolution were DMSO and chloroform. The data obtained were summarized in Tables 5A,B. The amide bond with UAconfirms the formation of ligand, which appears at 179 chemical shift. The peaks of metal complexes were detected between 9 and 10. The presence of OH in all metal complexes has been confirmed by 2–3 peaks. The C-28 and phenyl carbon in diphenyl tin complex have been presented by the peaks at 179 and 154 chemical shifts respectively as shown in Figure 6.

**Table 5A:** ^13^CNMR spectral data of ligand and their metal (Cu/Sb/Zn/Fe) complexes.

**Compound**		**UA**	**Ligand (L)**	**LFeCl_2_**	**LCu(Ac)_2_**	**LZn(Ac)_2_**	**LSbCl_2_**	**LSbBr_2_**
**C No**.	**Atom**	**δ(ppm)**	**δ(ppm)**	**δ(ppm)**	**δ(ppm)**	**δ(ppm)**	**δ(ppm)**	**δ(ppm)**
1	CH_2_	38	39.97	39.5	39.5	39.7	39.90	39.03
2	CH_2_	27.7	27.24	27.1	27.45	27.4	27.6	27.82
3	CH	78.5	80.3	78.7	80.64	80.6	78.61	79.62
4	C	39.5	40.94	40.5	41.42	40.4	40.37	41.4
5	CH	55.9	54.33	54	53	54.4	54.26	55.8
6	CH_2_	19	20.9	20	20.8	20.9	20.8	20.9
7	CH_2_	34.3	35.35	35.3	35.5	35.1	35.07	36.09
8	C	39.8	39.70	40	40.7	40.9	40.9	40.92
9	CH	48.5	47.81	48	48.6	47.7	48.84	49.55
10	C	37.5	37.1	38.5	38.1	38.08	38.03	39.04
11	CH_2_	25.7	25.7	25	25.07	25	25.82	25.83
12	CH	121.6	118.1	118.9	118.97	118.9	118.97	118.98
13	C	145.8	150.7	150.2	150.2	150.2.4	150.2	152.26
14	C	42.5	42.5	42.5	42.72	42.5	42.72	41.93
15	CH_2_	28.5	29.4	29.6	29.65	29.6	29.95	29.66
16	CH_2_	23.7	28.8	28.6	28.09	28.1	28.06	27.07
17	C	45.7	44.7	45	45.08	44.5	45.71	45.37
18	CH	42	54.0	54	54.7	54.6	55.5	54.6
19	CH	46.0	39.5	39	40.3	39.5	40.56	41.57
20	CH	30.5	38.1	38.8	39.6	39.9	39.83	38.84
21	CH_2_	34.5	29.31	30	29.4	30.4	29.3	28.93
22	CH_2_	33.5	35.7	33.7	34.5	33.7	35.33	35.34
23	CH_3_	28.9	18.8	19.5	18.9	18.9	18.9	18.92
24	CH_3_	17.6	19.5	19.5	19.0	19.0	19.0	19.47
25	CH_3_	20.6	22.66	21.7	20.5	21.7	20.5	21.56
26	CH_3_	17.5	20.6	18.9	19.5	19.0	19.2	18.9
27	CH_3_	26.5	25	21	21	21	21.0	26.07
28	C	180.2	180	181	180	180	178.0	179.08
29	CH_3_	33.5	16.7	16.7	17.7	17.5	17.00	18.0
30	CH_3_	21.8	18.81	19	18.6	19.5	19.02	20.0
31	C				175			
32	CH_3_				23.0			

**Table 5B:** ^13^CNMR spectral data of ligand and their metal (Tin) complexes.

**Compound**		**L**	**Ph_3_SnL**	**Ph_2_SnL_2_**	**Bu_3_SnL**	**Bu_2_SnL_2_**	**Me_3_SnL**	**Me_2_SnL_2_**
**C No**.	**Atom**	**δ(ppm)**	**δ(ppm)**	**δ(ppm)**	**δ(ppm)**	**δ(ppm)**	**δ(ppm)**	**δ(ppm)**
1	CH_2_	33.97	34.30	33.0	33.08	33.19	33.01	33.18
2	CH_2_	27.24	27.24	27.25	27.26	27.05	27.04	27.69
3	CH	80.3	77.09	77.39	77.35	77.09	80.33	79.73
4	C	40.94	41.91	40.93	41.95	41.56	41.03	39.11
5	CH	54.33	45.93	45.33	54.35	53.37	53.32	53.34
6	CH_2_	20.9	17.91	21.92	20.01	20.91	20.01	20.4
7	CH_2_	35.35	35.37	39.38	33.04	35.34	35.32	35.30
8	C	39.70	39.92	39.93	39.94	39.93	39.07	39.93
9	CH	47.81	47.81	47.82	47.85	47.85	47.40	47.65
10	C	37.1	39.8	39.82	37.98	37.82	37.90	37.96
11	CH_2_	25.7	25.78	21.79	25.77	25.78	25.74	25.67
12	CH	118.1	115.64	118.05	118.65	118.06	118.91	118.9
13	C	152.7	144.2	137.1	150.12	144.28	144.20	152.12
14	C	42.5	45.50	40.70	42.5	42.70	42.67	42.95
15	CH_2_	29.4	29.30	29.35	29.3	29.46	29.63	29.43
16	CH_2_	28.8	28.90	29.91	28.93	28.98	28.96	28.6
17	C	44.7	45.03	45.04	44.04	44.07	44.02	44.01
18	CH	54.0	61.00	61.19	54.2	52.21	54.17	54.95
19	CH	39.5	39.03	39.04	39.53	39.45	39.42	39.79
20	CH	38.1	39.80	39.81	39.91	38.51	38.49	38.88
21	CH_2_	29.31	29.40	29.45	29.5	29.54	29.51	29.54
22	CH_2_	35.7	33.71	33.14	33.75	33.14	33.71	33.70
23	CH_3_	18.8	18.95	18.51	18.92	19.01	19.50	18.69
24	CH_3_	19.5	19.49	19.55	19.35	19.60	19.50	19.57
25	CH_3_	22.66	15.60	21.50	21.53	25.22	21.70	21.63
26	CH_3_	20.6	18.31	18.34	18.4	18.45	18.42	18.44
27	CH_3_	25	21.00	21.02	21.0	25.2	21.19	21.12
28	C	180	152	152.71	178.3	152	180.9	179
29	CH_3_	16.7	16.72	16.74	16.95	16.89	16.74	16.30
30	CH_3_	18.81	18.91	18.80	18.9	18.4	18.90	18.86
1	C		137.67	137.67	8.3	11.10		−1.4
2	CH		128.30	128.30	26.3	23.40		−1.4
3	CH		129.3	129.36	13.8	13.80		
4	CH		128.2	128.22				
5′	CH		128.2	128.22				
6	CH		136.8	136.8				

### Biological assays

#### Antibacterial activity

For the screening of antibacterial activity, Agar Well Diffusion assay was carried out. The Tetracycline was used as positive control in the study. The results of antibacterial assay have been presented in Table 6. Synthesized ligand (UL) has shown lowest activity with MIC value of128 μg/mL against all studied strains. The antibacterial activity of metal complexes was recorded higher as compared to the ligands. Triphenyl tin complex (LTP) emerged as potent antibacterial agent with MIC value of 8 μg/mL each against *Shigellaspp, S. typhi*, and *S. aureus*. While, the MIC value against *Streptococcus pneumoniae* is 4 μg/mL. Similarly, tributyl tin complex (LTB) also showed excellent activity against Gram-positive species *S. aureus* and *S. pneumoniae* (8 μg/mL each, Table 6). The MIC value of LTB against Gram-negative strain (*Shigellaspp* and *S. typhi*) is16 μg/mL. Diphenyl tin complex (LDP) showed MIC value of 8 μg/mL for each strain. Dibutyl tin (LDB) and dimethyl tin (LDM) complexes also showed good activities. LDB with MIC value of 8 μg/mL exhibited excellent activity against *S. pneumoniae*. However, copper and zinc complexes were not able to show good activities. Two antimony complexes have shown the activity to some extent. Bromo antimony complex exhibited MIC value of 32 μg/mL against each strain.

**Table 6:** MIC values of synthesized metal complexes.

**Compound No**.	**Antibacterial activity (MIC in μg/ml)^a^**			
	***Shigella***	***S. typhi***	***S. aureus***	***S. pneumonia***
UL	>256	>256	>256	>256
LCu	>256	>256	128	>256
LZn	128	>256	>256	128
LFe	32	64	32	32
LSC	32	32	64	32
LSB	32	32	32	32
LDM	32	32	16	16
LTM	32	32	32	32
LDB	16	16	16	16
LTB	16	16	**8**	**8**
LDP	**8**	**8**	**8**	**8**
LTP	**8**^b^	**8**	**8**	**4**
Ciprofloxacin	8	NP^c^	8	NP
Penicillin V	0.01	0.01	0.05	0.05

a *The reported MIC values in μg/ml are an average of at least three individual measurements*.

b *The values shown in bold are the better/equal inhibitors than the standard drugs used*.

c *NP, Not performed*.

#### Antioxidant activity

**Figure 7:**
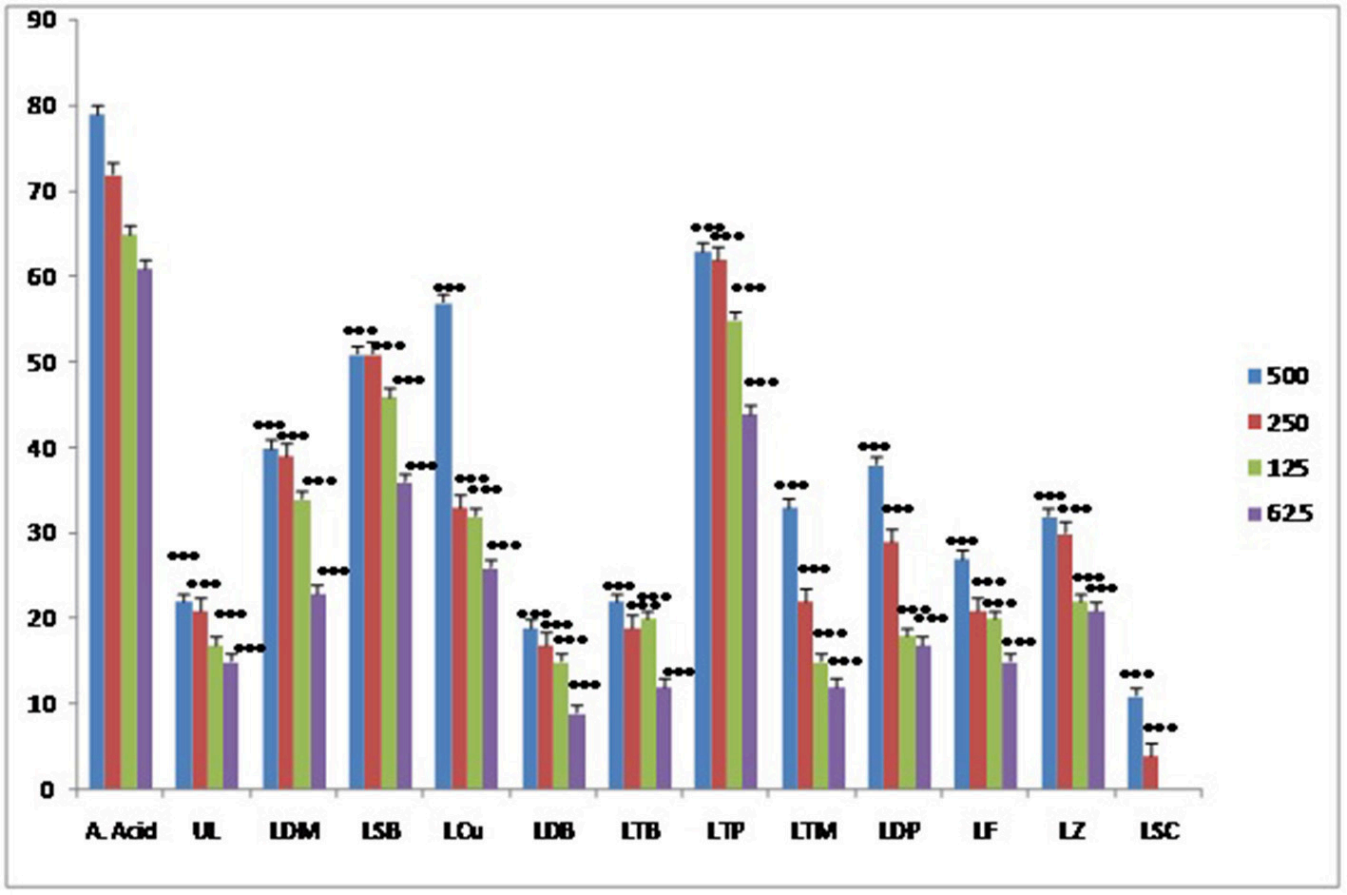
Results of antioxidant activity of various samples.

The antioxidant activity shown by the metal complexes and ligands were recorded as significant. In this assay ascorbic acid was employed as positive control. The radicals scavenging activity of metal complexes was recorded as higher as compared to the ligands. The highest antioxidant activity has been shown by the triphenyl tin complex as shown in the Figure 7. Similarly, the least antioxidant activity has been revealed by the antimony trichloride complex as the activity of this complex come to the baseline i.e., zero at 125 μg/ml.

Various compounds, either natural or synthetic are inevitable for the life of human on this planet. The compounds which are bestowed by the nature are not in the final shape (Leplege and Hunt, 1997). These are given in the humans' hands to manipulate and employ in its exact template. The compound UA possess a wide range of biological potential but this is not the final shape of UA to be employed. It should be transformed with various angles to get an appropriate template for a specific diseased condition. A myriad of compounds have been reported to possess negligible activities alone while in complex form their activities have been dramatically enhanced due to the action of various metals and other functional groups (Bruijnincx and Sadler, 2008). No doubt, the metals present in our body are extremely important in the terms of various metabolic processes in our body (Zatta et al., 2003). Similarly, if the activity of a nucleus enhances with the addition of certain metals then the same tactics should be applied to number of compounds so that no nucleus and no complex go unexplored. A similar attempt has been followed in the current study which has been proved to be a triumph by the results of biological activities. Only two biological activities were found out i.e., antibacterial and antioxidant, which are considered to be significant in comparison with the parent compound. These complexes are further under investigations in our labs for various biological potentials including, *in-vivo, in-vitro*, and *in-silico* studies. But the current research project may be a milestone the rest of the studies.

The metal complexes are potential for biological activities (Tarafder et al., 2001). The metal complexes of various compounds have been evaluated for different activities, especially as antimicrobials (Chohan et al., 2004; Daniel et al., 2008). Bravo and co-workers have shown that the metal complexes of quercetin possess strong antibacterial activity (Bravo and Anacona, 2001). The metal complexes of coumarins and its derivatives are potential agents against various bacterial and fungal strains (Rehman et al., 2005). The tin complexes of different compounds are potential complexes in the management of leishmaniasis, free radicals scavenging, DNA interaction and as antimicrobial (Raychaudhury et al., 2005; Raza et al., 2016). The complexes of antimony have also a vital role in antimicrobial, antioxidant and anticancer activities (Viswanathan et al., 2000; Ejidike and Ajibade, 2015; Neelofar et al., 2016). The zinc complexes of several different classes of compounds have been extensively studied as an antioxidant and antimicrobial agents (Howard et al., 1973; Wang et al., 2006; Kadhum et al., 2011; Zaidi et al., 2012; Sheikh et al., 2013; Tarushi et al., 2013; Urquiza et al., 2013; Wu et al., 2013; Montazerozohori et al., 2014; Ebrahimiasl et al., 2015). As compared to other metal complexes, our synthesized derivative and its metal complexes have shown prominent antioxidant and antibacterial activities (Kadhum et al., 2011; Sadiq et al., 2017).

### Docking studies

#### Docking studies on antioxidant enzyme peroxiredoxins (Prxs)

In current study, antioxidant activity was determined by DPPH free radical method. However, we tried to interpret the antioxidant results at molecular level. Antioxidant assay revealed that that triphenyl tin complex exhibited significant activity. Here, we tried to interpret the experimental results with the binding mode comparison of the synthesized compounds by carrying out the docking studies against human antioxidant enzyme. We have selected peroxiredoxins (Prxs) for the interpretation of our results. Peroxiredoxins (Prxs) are thiol dependent antioxidant enzymes that reduce hydrogen peroxide and alkyl peroxides to water and alcohol (Hall et al., 2010).

**Figure 8:**
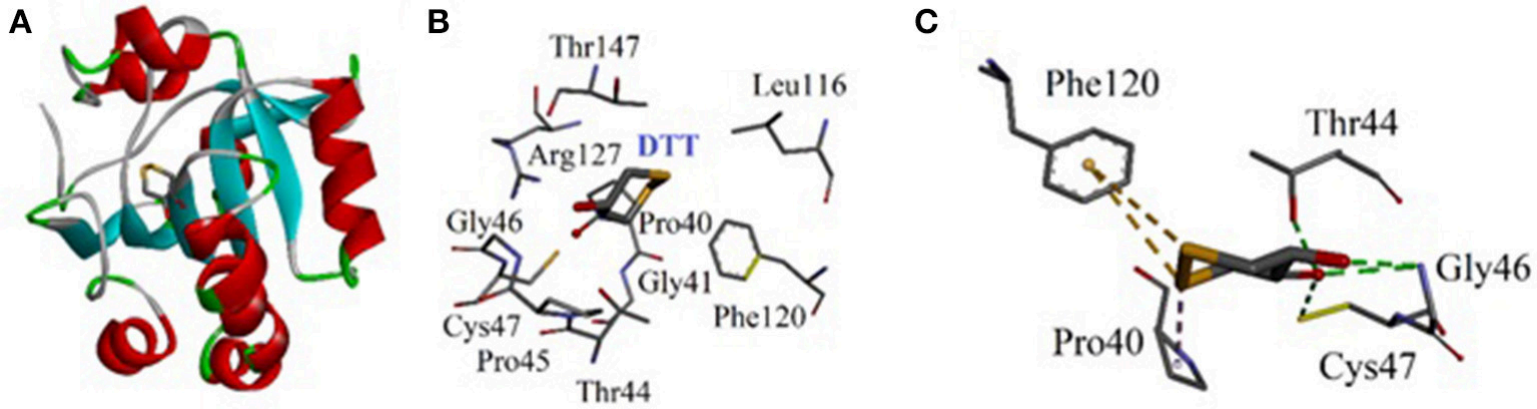
**(A)** Computer generated structure ofhuman PrxV (PDB code 3MNG) with a competitive antioxidant inhibitor 1,2-dithiane-4,5-diol (DTT) (ribbon form); **(B)** Active site of 3MNG; **(C)** Interactions of DTT with important residues.

Molecular docking studies were carried out on using Autodock 4.2. The three dimensional structure of human PrxV (PDB code 3MNG) with a competitive antioxidant inhibitor 1,2-dithiane-4,5-diol (DTT) was retrieved from Protein Data Bank (PDB). The binding orientation of the co-crystallized ligand is shown in Figure 8A. The active site of the enzyme consists of Pro40, Gly41, Thr44, Pro45, Gly46, Cys47, Leu116, Phe120, Arg127, and Thr147 (Figure 8B). Cys47 is considered as an essential residue for binding (Hall et al., 2010). DTT established four strong hydrogen bonding interactions. A bifurcated hydrogen bond is established between two hydroxyl groups of DTT and Gly46. Thr44 and Cys47 also forms hydrogen bonding interactions with DTT hydroxyl groups (Figure 8C).

**Figure 9:**
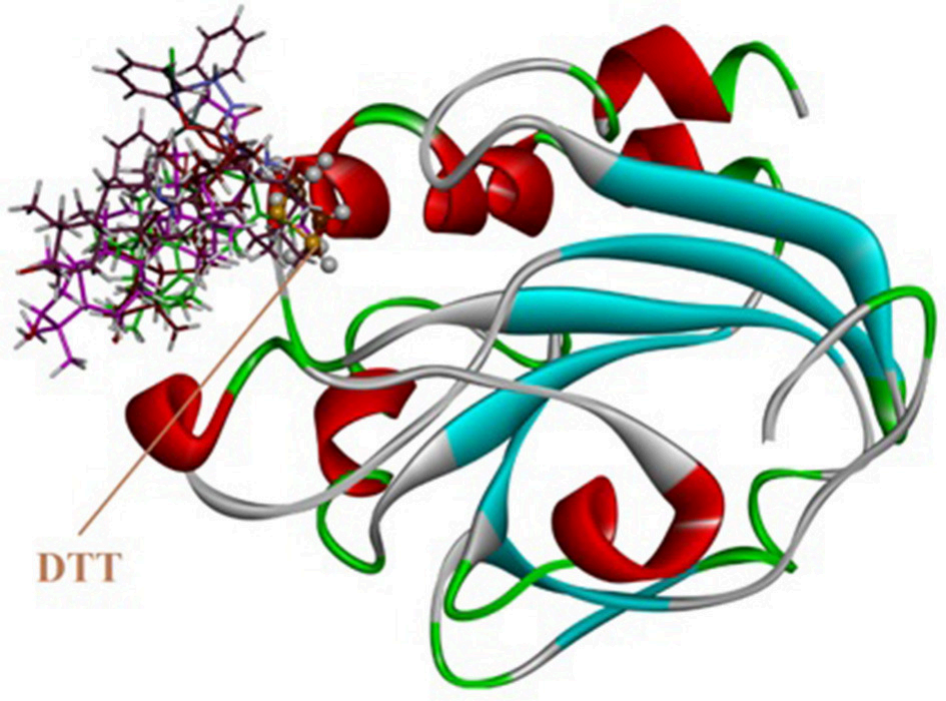
Superposed 3D modeled diagram of some metal complexes into the active site of 3MNG.

**Figure 10:**
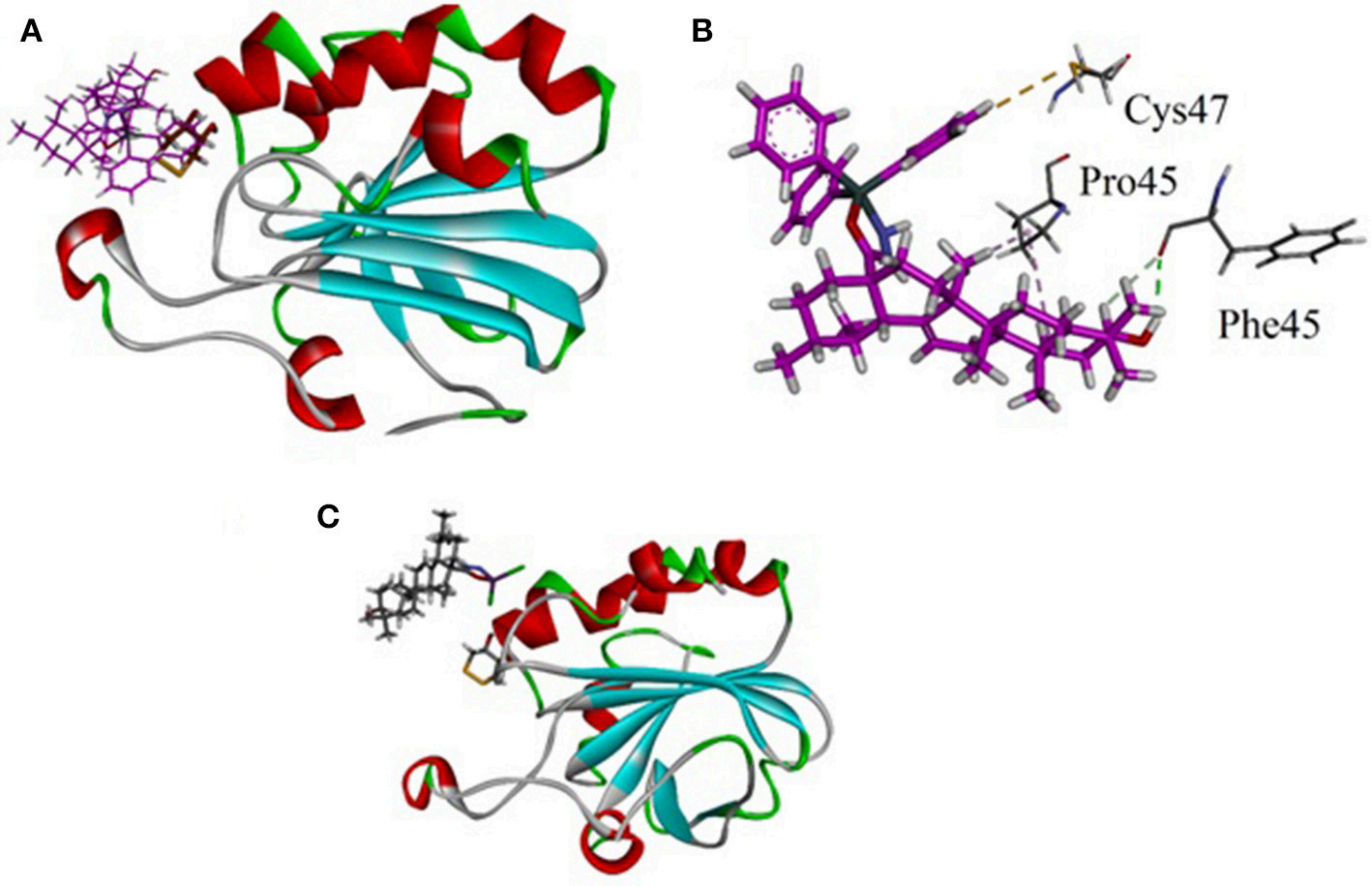
**(A)** Lowest energy bonding pose of LTP superposed on DTT into the binding site of 3MNG; **(B)** Close-up view showing molecular interactions exhibited by LTP (purple); **(C)** The binding mode of LSC exhibited poor antioxidant activity.

The synthesized metal complexes were docked into the active site of 3MNG. Superposed 3D modeled diagram of some metal complexes is shown in Figure 10. Figure 9 showed that not all he complexes are bounded into the binding site of the competitive inhibitor DTT.

As described above triphenyl tin complex (LTP) showed highest antioxidant activity. The computer generated 3D modeled docked diagram of the LTP showed that it is bounded into the DTT binding site (Figure 10A). Furthermore, it also shows π-sulfur interactions with catalytically important residue Cys47. A hydrogen bonding interaction was also shown by LTP and Phe45. Some hydrophobic interactions were also observed between the complex and Pro45 (Figure 10B). The estimated Free Energy of Binding of the compound is −4.34 kcal/mol. Antimony trichloride complex (LSC) was found least active complex with antioxidant activity zero at 125 μg/ml. The binding mode of LSC is shown in Figure 10C. It is revealed from Figure 10C that LSC bounded away from DTT and have shown no interactions with catalytically important amino acid residues.

#### Docking studies on antibacterial target protein binding penicillin

Bacterial cell wall is a validated target for the drug discovery researchers because the enzyme involved in its synthesis has no counterpart in mammalians. Penicillin antibiotics area major class that targets the bacterial cell wall (Sadiq et al., 2017). The standard drug used in our antibiotic study was penicillin V also known as phenoxymethylpenicillin. Penicillin V, like other penicillin derivatives, inhibits the cell wall peptidoglycan.

**Figure 11:**
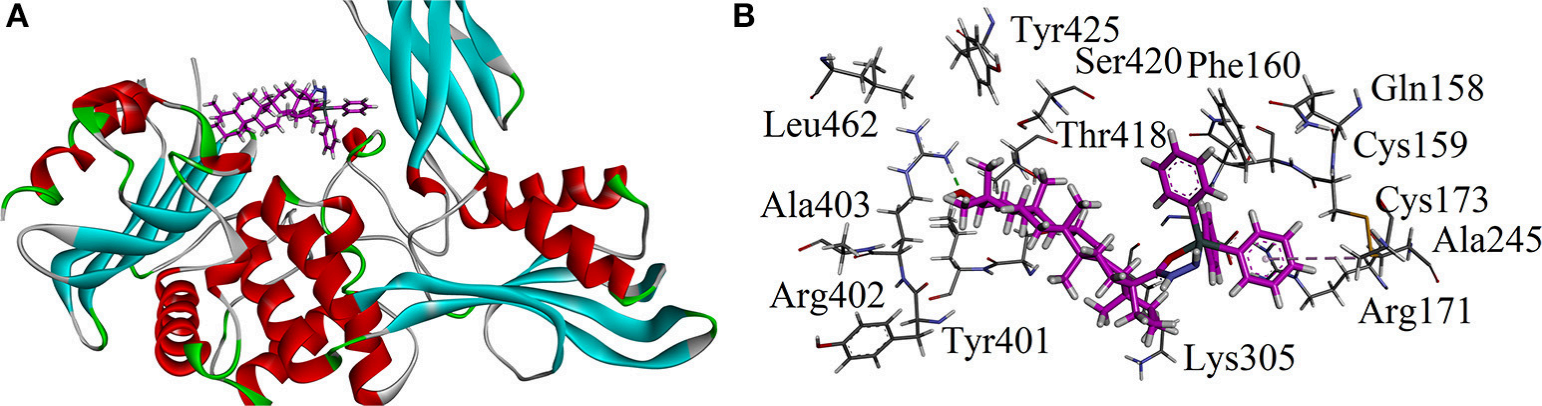
**(A)** The three dimensional (3D) of triphenyl tin complex (LTP) into the binding site of 2EX9; **(B)** Close-up depiction of docking pose of LTP showing interactions.

Keeping in view the mechanism of action of standard drug used, we carried out docking studies on penicillin binding protein (PBP). Crystal structure of PBP in complex with penicillin V was retrieved from Protein Data Bank (PDB code 2EX9). The three dimensional (3D) of triphenyl tin complex (LTP) is shown in Figure 11A. The important amino acid residues are shown in Figure 11B. Compound LTP showed one hydrogen binding interactions with Arg402 and an hydrophobic interaction with Ala245. The estimated Free Energy of Binding of the LTP is −3.98 kcal/mol.

## Conclusion

Based on the findings of our current studies, it may be inferred that the activity of ursolic acid can be enhanced by making complexes with various metals. It may also be deduced that certain metal complexes of ursolic acids have been verified as strong antibacterial and antioxidant than ursolic acid alone, which should be further verified and screened for other assays so that they may be proved for specific potential, which can open the way to the market. Binding mode analysis has shown a fair correlation between experimental study and estimated energy of binding.

## Author contributions

MJ and KS carried out the experimental work. SA and MJ drafted the manuscript. UR performed the docking studies. AS refined the manuscript.

### Conflict of interest statement

The authors declare that the research was conducted in the absence of any commercial or financial relationships that could be construed as a potential conflict of interest.
