# Corrigendum: Ursolic Acid Hydrazide Based Organometallic Complexes: Synthesis, Characterization, Antibacterial, Antioxidant, and Docking Studies

**DOI:** 10.3389/fchem.2018.00343

**Published:** 2018-08-14

**Authors:** Muafia Jabeen, Sajjad Ahmad, Khadija Shahid, Abdul Sadiq, Umer Rashid

**Affiliations:** ^1^Department of Pharmaceutical Chemistry, Riphah Institute of Pharmaceutical Sciences, Riphah International University, Islamabad, Pakistan; ^2^Department of Pharmacy, University of Malakand, Chakdara, Pakistan; ^3^Department of Chemistry, COMSATS Institute of Information Technology, Abbottabad, Pakistan

**Keywords:** ursolic acid, triterpenoid, metal complex, hydrazine, antioxidant, antibacterial, molecular docking

In the published article, there was an error in affiliation 1. Instead of “Department of Pharmacology, Riphah Institute of Pharmaceutical Sciences, Riphah International University, Islamabad, Pakistan”, it should be “Department of Pharmaceutical Chemistry, Riphah Institute of Pharmaceutical Sciences, Riphah International University, Islamabad, Pakistan”. The authors apologize for this error and state that this does not change the scientific conclusions of the article in any way.

The original article has been updated.

## Conflict of interest statement

The authors declare that the research was conducted in the absence of any commercial or financial relationships that could be construed as a potential conflict of interest.

